# A Comprehensive Study on the Mechanistic Way of Hexaflumuron and Hymexazol Induced Neurobehavioral Toxicity in Rats

**DOI:** 10.1007/s11064-022-03654-5

**Published:** 2022-06-30

**Authors:** Eman I. Hassanen, Ahmed M. Hussien, Neven H. Hassan, Marwa A. Ibrahim, Sally Mehanna

**Affiliations:** 1 Department of Pathology, Faculty of Veterinary Medicine, Cairo University, P.O. Box 12211, Giza, Egypt; 2 Department of Toxicology and Forensic Medicine, Faculty of Veterinary Medicine, Cairo University, Giza, Egypt; 3 Department of Physiology, Faculty of Veterinary Medicine, Cairo University, Giza, Egypt; 4 Department of Biochemistry and Molecular Biology, Faculty of Veterinary Medicine, Cairo University, Giza, Egypt; 5 Department of Veterinary Hygiene and Management, Faculty of Veterinary Medicine, Cairo University, Giza, Egypt

**Keywords:** Behavioral changes, Hexaflumuron, Hymexazol, Neurotoxicity, Pathology

## Abstract

Pesticides are widely used in agriculture to kill pests, but their action is non-selective and results in several hazardous effects on humans and animals. Pesticide toxicity has been demonstrated to alter a variety of neurological functions and predisposes to various neurodegenerative diseases. Although, there is no data available for hexaflumuron (HFM) and hymexazol (HML) neurotoxicity. Hence, the present study aims to investigate the possible mechanisms of HFM and HML neurotoxicity. 21 male Wistar rats were divided into three groups and daily received the treatment via oral gavage for 14 days as follows: group (1) normal saline, group (2) HFM (1/100LD50), and group (3) HML (1/100 LD50). Our results revealed that both HFM and HML produced a significant increase in MDA levels and a decrease in GSH and CAT activity in some brain areas. There were severe histopathological alterations mainly neuronal necrosis and gliosis in different examined areas. Upregulation of mRNA levels of JNK and Bax with downregulation of Bcl-2 was also recorded in both pesticides exposed groups. In all studied toxicological parameters, HML produced neurotoxicity more than HFM. HFM targets the cerebral cortex and striatum, while HML targets the cerebral cortex, striatum, hippocampus, and cerebellum. We can conclude that both HFM and HML provoke neurobehavioral toxicity through oxidative stress that impairs the mitochondrial function and activates the JNK-dependent apoptosis pathway.

## Introduction

Much progress has been made in identifying genes involved in the familial forms of different neurodegenerative and neuroinflammatory disorders. However, the majority of disease cases are sporadic, and their origin remains largely unidentified. The environment is a key contributor to human health and disease and plays a role in the etiology of neurodegenerative diseases. Ever-increasing human impact on nature presents an urgent issue of environmental conservation. The overuse of pesticides in agriculture leads to the deposition of such harmful chemicals in soil, water basins, and agricultural runoff [1]. Pesticides are chemicals that are commonly used to manage pests like insects, weeds, fungi, and rodents. Most pesticides aren’t very selective, and they're dangerous to non-target animals like humans and animals. Neurotoxicity can be caused by a variety of pesticides. A thorough understanding the mechanism of action of hazardous chemicals at the cellular and subcellular levels can be suggested as appropriate nature-conserving strategies. Most insecticides kill insects by attacking their nervous systems, therefore it can also cause neurotoxicity in mammals [2].

Hexaflumuron (HFM) is a systemic insect growth regulator (IGR) from the benzoylurea’s family of pesticides. It works by blocking the chitin synthesis in the insect's body. Otherwise, this insecticide is hazardous and can be absorbed through the skin, respiratory, and digestive systems of humans [3]. Headache, dizziness, and nausea are some of the harmful consequences of HFM, which are common in farmers who are exposed to it on the job [4]. Hexaflumuron has a rat LD50 of over 5000 mg/kg and is considered a low-toxic chemical [5]. A recent study revealed the toxic impact of HFM on the liver and kidney tissues of rats [6], while other studies report the immune toxic effect of HFM in rats and other experimental animals [7].

Hymexazol (HML) is a heteroaryl hydroxy chemical that is used as a systemic fungicide to treat soil and seed fungal infections. Seed treatment with HML at 10.5–14 g/kg seed was recommended by Payne and William [8]. However, it is highly toxic to embryo fish and can cause cardiac edema, growth retardation, and swing abnormalities [9]. European Food Safety Authority (EFSA) has identified the HML hymexazol as a toxic agent for humans. It is reported that the liver is the main target organ after short-term repeated exposure to HML in rats [10]. In spite of increasing the use of these pesticides in the developing countries in the agricultural and veterinary practices, there isn’t much data about its toxicity on the brain. Therefore, the present study aims to elucidate the possible mechanism of HFM- and HML-induced neurotoxicity in rats by studying the neurobehavioral changes, oxidative stress evaluations, and histopathological examination of some brain areas including the cerebrum cortex, striatum, hippocampus, and cerebellum. Additionally, we study the effect of both pesticides on the JNK signaling pathway.

## Materials and Methods

### Chemicals

Marketed hexaflumuron (10% active ingredient, EC, 1-[3,5-dichloro-4-(1,1,2,2-tetrafluoroethoxy)phenyl]-3-(2,6-difluorobenzoyl)urea) and marketed hymexazol (30% active ingredient, EC, 5-methylisoxazol-3-ol) were obtained from the Advanced Co. for Agricultural Pesticides industrial production, Jordan. These pesticides were freshly prepared in deionized distilled water according to the required concentration of the active ingredient.

### Animals and Experimental Design

Twenty-one male albino Wistar rats (170–20 g) were purchased from Cairo University, Faculty of Veterinary Medicine, Department of Veterinary Hygiene and Management’s Animal House. Animals were raised in plastic cages, fed commercial pelleted feed, and given free access to water. Before being used, they were assessed for health and acclimatized to the research laboratory setting for 2 weeks. The Cairo University’s institutional animal care and use committee (IACUC) approved all the procedures and experimental design (approval number: Vet CU12102021361).

Rats were randomly divided into three groups (n = 7) and were given the following materials every day by oral gavage for 14 days. Group (1) received normal saline and was kept as a control negative group. Group (2) received HFM at 50 mg active ingredient/kg bwt representing 1/100 LD50. Group (3) received HML at 39 mg active ingredient/kg bwt representing 1/100 LD50. Doses of pesticides were selected based on their LD50 which was reported to be 5000 mg/kg bwt for HFM**,** and 3900 mg/kg bwt for HML [11].

All rats were observed daily during the experimental period for any clinical signs including awareness, motor activity, and posture abnormalities. Furthermore, body weight was recorded at the onset of the treatment as well as at the end of the study.

### Behavioral Evaluations

#### Light–Dark Transition Task

The light–dark test is one of the simplest and most well-known tests used for the assessment of anxiety and emotionality. The apparatus was made up of an open-topped, light chamber connected to a closed-topped, dark chamber, each chamber comprising (30 × 40 × 40 cm). A small aperture connects the chambers, allowing the rat to move between them. The rat was situated in the lightbox and permitted to roam freely between the chambers for 5 min while its location was observed. The duration spent on the light side of this device throughout the 5 min test session compared to the dark side was recorded and utilized as an indication of anti-anxiety behavior [12].

#### Rod Walking Test

Psychomotor coordination is measured by rats' ability to balance on a stationary horizontal rod. Rats were positioned in the middle of a horizontal elevated rod (100 cm length, 2.6 cm diameter). The time of falling of the rat into a cushion placed below was measured with a limited time of 60 s [13].

#### Object Recognition Test

The object recognition test is a popular behavioral experiment for studying several aspects of learning and memory in rats and mice. In the back left and right corners of an empty cage, two identical objects were placed. The rat was positioned in the center of the cage, opposite the objects. The rat was allowed to investigate these objects for 10 min before being taken from the cage and returned to its colony for 1 h. To assess for object recognition, one of the familiar objects was set in one back corner of the cage, and a novel object was set in the other back corner, and the rat was placed in the same manner as previously mentioned for 4 min. The rat’s time spent exploring each object was measured [14].

### Sampling

After the behavioral assessments, rats were anesthetized with single intramuscular injections of xylazine and ketamine and euthanized by cervical dislocation to collect the whole brain that was divided into different areas (cerebrum cortex, striatum, hippocampus, and cerebellum). Part of these areas was stored at − 80 °C till used for oxidative stress evaluation and molecular studies, while others were preserved in 10% neutral buffered formalin to perform the histopathological examinations.

### Oxidative Stress Evaluation

We homogenized all the collected specimens obtained from different brain areas by using cold PBS (50 mM potassium phosphate buffer containing 1 mM EDTA, PH 7.4) and centrifuged them at 4500 rpm for 5 min to collect the supernatant that was used for further oxidative stress marker evaluations. Malondialdehyde (MDA), reduced glutathione (GSH), and catalase (CAT) were measured according to the instructions of the manufacturer kits (Biodiagnostic Com., Cairo, Egypt).

### Quantitative RT-PCR Analysis of JNK, Bcl2, and Bax Genes

Total RNA from various tissues was isolated using the RNeasy Mini Kit (Qiagen Cat No./ID: 74104). First-strand cDNA synthesis was performed using Superscript reverse transcriptase according to the manufacturer’s instructions. Table 1 shows the primer set used to amplify the target genes. Quantitative PCR was performed using the SYBR™ Green PCR Master Mix (Thermoscientific Cat #: 4309155) [15]. ABI Prism Step One Plus Real-Time PCR System & # 40; Applied Biosystems & # 41; According to manufacturer information. The assay was performed in triplicate and normalized using ACTB [16].

**Table 1:** The primer sets for the studied genes

Gene	Forward primer	Reverse primer	Product	Accession no.
JNK	GTCATTCTCGGCATGGGCTA	TGGACGCATCTATCACCAGC	337	NM_053829.2
*Bax*	CACGTCTGCGGGGAGTCAC	TTCTTGGTGGATGCGTCCTG	248	NM_017059.2
*Bcl-2*	TCGCGACTTTGCAGAGATGT	CAATCCTCCCCCAGTTCACC	116	NM_016993.2
*ACTB*	CCGCGAGTACAACCTTCTTG	CAGTTGGTGACAATGCCGTG	297	NM_031144.3

*JNK* c-Jun N-terminal Kinase; *Bax* B-cell lymphoma 2 associated X protein; *Bcl2* B-cell lymphoma 2; *ACTB* beta actin housekeeping gene

### Histopathological Examination

We conventionally processed the formalin‐fixed brain tissue samples utilizing ascending grades of alcohol and xylene. Afterward, we embedded them in paraffin wax and sliced them by the ordinary microtome at 4.5 μm, then stained sections with H&E. All stained sections were examined under light Olympus BX43 microscope and captured images by Olympus DP27 camera linked with CellSens dimension software XV3.12 (version 1.13) [17].

The observed microscopic lesions in different brain areas were semi-quantitatively scored according to the method described by Hassanen et al., [18]. The diffuse microscopic changes as neuronal degeneration and necrosis, diffuse gliosis, and vascular congestion were graded and scored as slight, mild, moderate, and severe as follows (0 = normal histology, 1 < 25%, 2 = 25:50%, 3 = 50:75%, and 4 > 75% tissue damage). The shrunken neurons with pyknotic or completely lytic nuclei that are surrounded by numerous glia cells are considered necrotic and/or degenerated neurons. The grading scheme for any focal lesions such as focal gliosis and hemorrhage was assessed as follows: (0) no foci; (1) < 3 foci; (2) 3–6 foci; (3) 7–12 foci; (4) > 12 foci/field at low power (200×) [19].

Histomorphometric analysis was performed by using Image J software to determine the diameter of the neuronal cell body [(the maximum length + the maximum width)/2], the total number of neurons per mm^2^, and then we calculated the neuronal cell density in the examined brain areas according to the following equation:

$${\text{ND}} = {{{\text{NC}}} \mathord{\left/ {\vphantom {{{\text{NC}}} {{\text{A}}\left( {{\text{D}} + {\text{T}}} \right)}}} \right. \kern-\nulldelimiterspace} {{\text{A}}\left( {{\text{D}} + {\text{T}}} \right)}}$$

(ND) means neuronal cell density per mm^3^, (NC) means total neuronal count per mm^2^ in a selected square, (A) means the area of the square by mm^2^, (D) means the mean diameter of the neuronal bodies by mm, and (T) means the thickness of brain tissue in section by mm. Only the clear neurons with distinct vesicular nuclei were counted, while the shrunken darkly stained neurons with pyknotic or fragmented nuclei are excluded from the counting. All the morphometric analysis was blindly done using the same microscope and the same objective lens at a magnification of ×4000 in at least 7 fields/section in a total of 3 sections/group. Additionally, it was done within the same brain region and the same area in this region in all the treatment groups.

### Immunohistochemical Staining

Caspase-3 protein was localized within the cytosol of necrotic neurons using a casp-3 primary antibody and DAP Kit. Formalin‐fixed paraffin‐embedded sections were incubated with casp-3 primary antibody (Abcam, Ltd.) at 1/200 dilutions, then incubated with Peroxidase Block (Sakura BIO) and the reagent required for the identification of the antigen‐antibody complex (Power‐Stain 1.0 Poly HRP DAP Kit; Sakura). The sections were treated with 3,3′‐diaminobenzidine chromogen substrate for 10 min and counterstained with Hematoxylin and inspected by a light Olympus BX43 microscope, and caught images by Olympus DP27 camera connected to CellSens dimensions software (Olympus).

### Statistical Analysis

All data were expressed as means ± standard error of the mean (SEM) and analyzed by using SPSS version 24.0 software (SPSS Inc., Chicago, IL, USA). One-way analysis of variance (ANOVA) followed by Post-Hoc Duncan’s test was performed to compare the different groups. Whereas, the non-parametric values as neurobehavioral alterations were expressed as median and analyzed by using the Kruskal Wallis H test followed by the Mann–Whitney *U* test. Values of *P* ≤ 0.05 and *P* ≤ 0.01 were considered statistically significant.

## Results

### General Observation and Body Weight of Rats

**Fig. 1:**
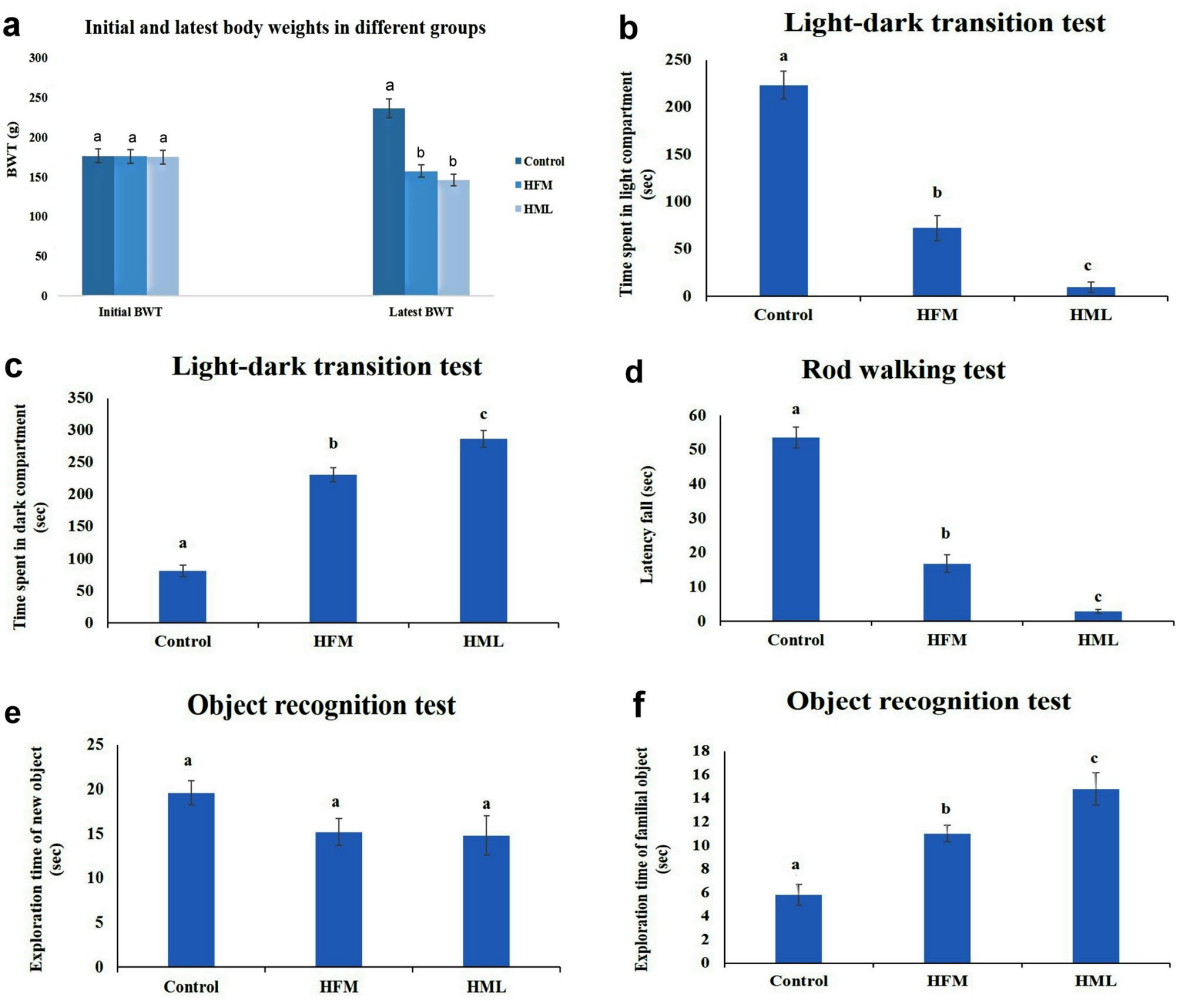
Bar charts representing the effects of HFM and HML on the body weights and some neurobehavioral test in rats. **a** Initial and latest body weight of rats. **b**, **c** Time (s) spent in the light and dark compartment respectively during the light–dark transition test. **d** Falling time (s) during the rod walking test. **e**, **f** Exploration time (s) of new and familiar objects respectively during the object recognition test. Values are presented as mean ± SEM (n = 7 rats/group). Values denoted with different superscript letter (a–c) are statistically significant at P ≤ 0.05

Exposure to HML decreased the general activity of all rats including dullness, incoordination, and lethargy, While HFM-exposed rats did not display any particular clinical signs. Regarding the body weight, rats given either HFM or HML had significantly lower body weights than those in the control group. Furthermore, when compared to the HFM-exposed group, HML caused a significant reduction in body weight (Fig. 1a).

### Behavioral Evaluation

#### Light–Dark Transition Task

Rats under HFM and HML effects significantly diminished the time spent in the light compartment, accompanied by a significant increase of this measure in the dark compartment compared to control rats. Furthermore, time spent by HML-exposed rats in the light compartment was significantly decreased, while significantly increased in the dark compartment compared to HFM-treated rats (Fig. 1b, c).

#### Rod Walking Test

Exposure to HFM and HML caused a significant decrease in falling time compared to the animals belonging to the control group. Moreover, HML-treated rats displayed a marked significant decrease in falling time in comparison to the HFM-exposed rats (Fig. 1d).

#### Novel Object Recognition Test

Administration of HFM and HML to rats produced a non-significant difference in the time required to explore a new object and a significant increase in the time required to explore a familiar object when compared to their control counterparts. In addition, rats administered HML significantly spent more time in the exploration of a familiar object in comparison to counterparts in the HFM group (Fig. 1e, f).

### Oxidative Stress Evaluation

Rats exposed to either HFM or HML showed a significant increase in MDA levels with a decrease in GSH and CAT activity in different brain areas compared to the control group. Additionally, rats exposed to HML displayed a significant increase in MDA levels and a decrease in GSH and CAT activity in comparison to the HFM-exposed rats (Table 2).

**Table 2:** The effect of hexaflumuron (HFM) and hymexazol (HML) on some oxidative stress markers in different brain regions

Markers/groups	Control	HFM	HML
MDA (nmol/mg)			
Cerebrum cortex	0.54 ± 0.04^a^	3.8 ± 0.43^b^	5.2 ± 0.28^c^
Striatum	0.72 ± 0.01^a^	1.6 ± 0.29^b^	2.4 ± 0.25^b^
Hippocampus	0.84 ± 0.04^a^	1.05 ± 0.18^a^	2.8 ± 0.36^b^
Cerebellum	1.02 ± 0.08^a^	4.7 ± 0.32^b^	9.5 ± 0.76^c^
GSH (mg/g)			
Cerebrum cortex	16.6 ± 1.02^a^	12.8 ± 0.54^b^	8.5 ± 0.35^c^
Striatum	16.2 ± 0.97^a^	13.4 ± 0.52^b^	6.6 ± 0.62^c^
Hippocampus	18.6 ± 1.07^a^	16.4 ± 0.74^a^	5.2 ± 0.73^b^
Cerebellum	14.2 ± 0.73^a^	13.2 ± 0.34^a^	4.4 ± 0.46^b^
TAC (nmol/mg)			
Cerebrum cortex	173.8 ± 6.4^a^	113.2 ± 5.1^b^	95.2 ± 3.2^c^
Striatum	162 ± 8.2^a^	141 ± 3.4^ab^	104.3 ± 3.4^b^
Hippocampus	192 ± 5.6^a^	190.4 ± 4.2^a^	96.8 ± 3.4^b^
Cerebellum	142 ± 3.7^a^	132.4 ± 2.8^a^	82.3 ± 2.2^b^

Values are presented as mean ± SEM (n = 7 rats/group). Values denoted with different superscript letter (a–c) in the same row are statistically significant at P ≤ 0.05

### Quantitative RT-PCR Analysis of JNK, Bax, and Bcl2 Genes

Administration of HFM and HML to rats produced a significant upregulation in the mRNA levels of JNK and Bax genes with downregulation in the mRNA levels of Bcl2 genes compared to the control group. Additionally, rats exposed to HML exhibit marked upregulation in the mRNA levels of JNK and Bax genes with downregulation in the mRNA levels of Bcl2 genes in comparison to the HFM-exposed rats (Table 3).

**Table 3:** The effect of hexaflumuron (HFM) and hymexazol (HML) on the mRNA levels of some genes in the brain tissue

Genes/groups	Control	HFM	HML
JNK (fold change)			
Cerebrum cortex	1 ± 0.00^a^	3.84 ± 0.26^b^	4.4 ± 0.16^c^
Striatum	1 ± 0.00^a^	3.53 ± 0.42^b^	4.63 ± 0.74^c^
Hippocampus	1 ± 0.00^a^	2.3 ± 0.21^b^	4.7 ± 0.33^c^
Cerebellum	1 ± 0.00^a^	2.51 ± 0.14^b^	4.51 ± 0.20^c^
Bax (fold change)			
Cerebrum cortex	1 ± 0.00^a^	5.64 ± 0.4^b^	6.4 ± 0.6^c^
Striatum	1 ± 0.00^a^	5.52 ± 0.2^b^	6.3 ± 0.4^c^
Hippocampus	1 ± 0.00^a^	4.53 ± 0.42^b^	6.23 ± 0.2^c^
Cerebellum	1 ± 0.00^a^	4.25 ± 0.33^b^	6.1 ± 0.34^c^
Bcl-2 (fold change)			
Cerebrum cortex	1 ± 0.00^a^	0.3 ± 0.06^b^	0.31 ± 0.12^b^
Striatum	1 ± 0.00^a^	0.3 ± 0.12^b^	0.31 ± 0.02^b^
Hippocampus	1 ± 0.00^a^	0.64 ± 0.02^b^	0.23 ± 0.01^c^
Cerebellum	1 ± 0.00^a^	0.62 ± 0.11^b^	0.22 ± 0.04^c^

Values are presented as mean ± SEM (n = 7 rats/group). Values denoted with different superscript letter (a–c) in the same row are statistically significant at P ≤ 0.05

### Histopathological Examination

**Fig. 2:**
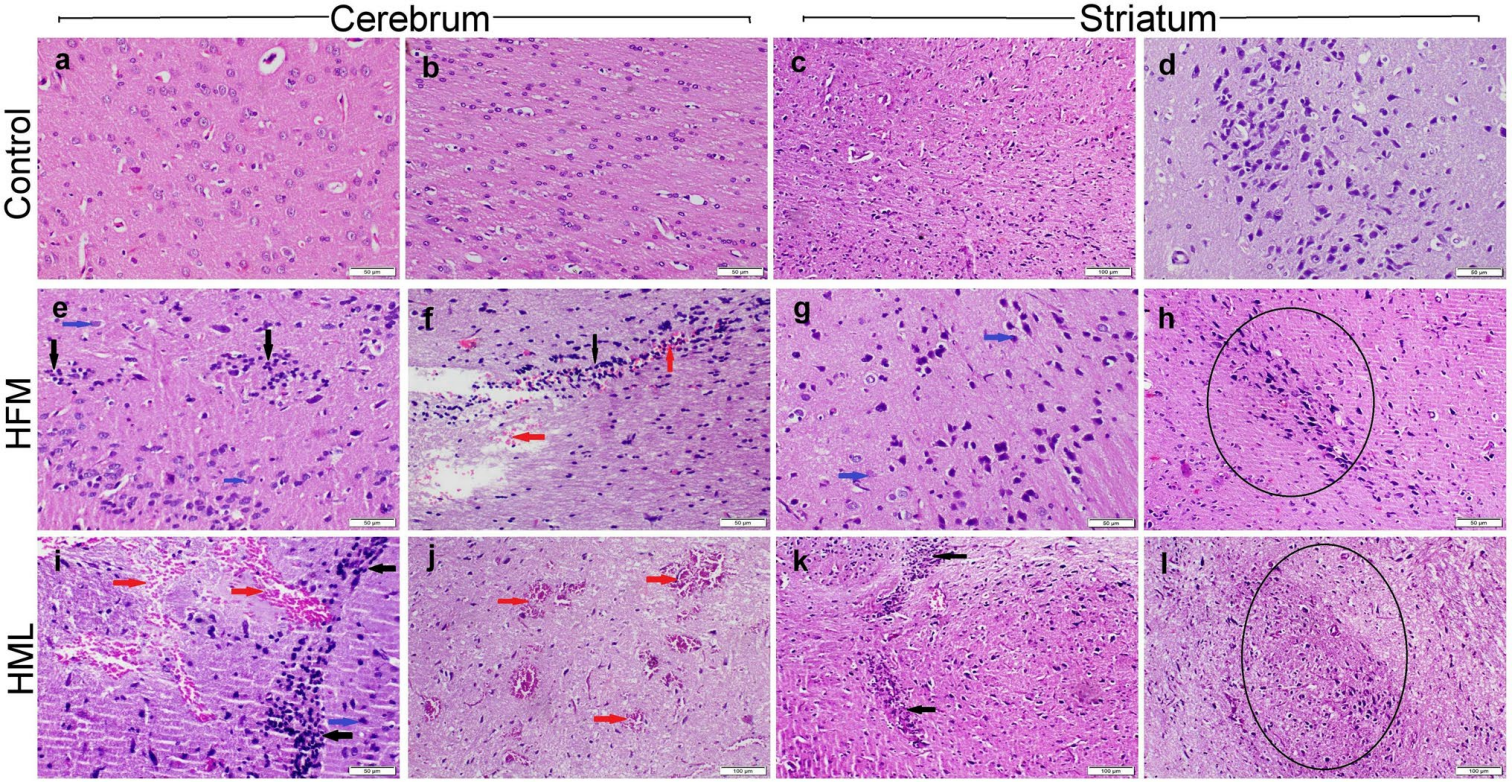
Photomicrograph of H&E staining sections representing the cerebral cortex and striatum of different treatment groups. **a**–**d** The control group showed normal histological organization. **e**–**h** HFM receiving group, and **i**–**l** HML receiving group. *Note* Neuronal degeneration (blue arrow), gliosis (black arrow), extravasated RBCs (red arrows), poorly identified substantia nigra (circle)

The control rats showed normal histological organization of different brain areas including the cerebrum cortex (Fig. 2a, b), and striatum (Fig. 2c, d), whereas rats exposed to either HFM or HML showed remarkable histopathological alterations in these brain areas. Concerning HFM-exposed rats, the cerebral cortex showed mild to moderate neuronal degeneration with neuronophagia (Fig. 2e). The grey matter showed focal to diffuse gliosis with vacuolation in the neuropil. White matter showed congestion, extravasation of erythrocytes, and focal gliosis (Fig. 2f). The striatum showed mild degeneration in both corticostriatal and nigrostriatal neurons, with moderate diffuse gliosis (Fig. 2g). Moreover, the substantia nigra didn’t clearly outline in most sections (Fig. 2h). Regarding HML exposed group, the microscopic lesions observed in the cerebral cortex (Fig. 2i, j) and striatum (Fig. 2k, l) were similar to those observed in the HFM group but more severe and accompanied by extensive hemorrhage and gliosis either in the focal or diffuse patterns.

**Fig. 3:**
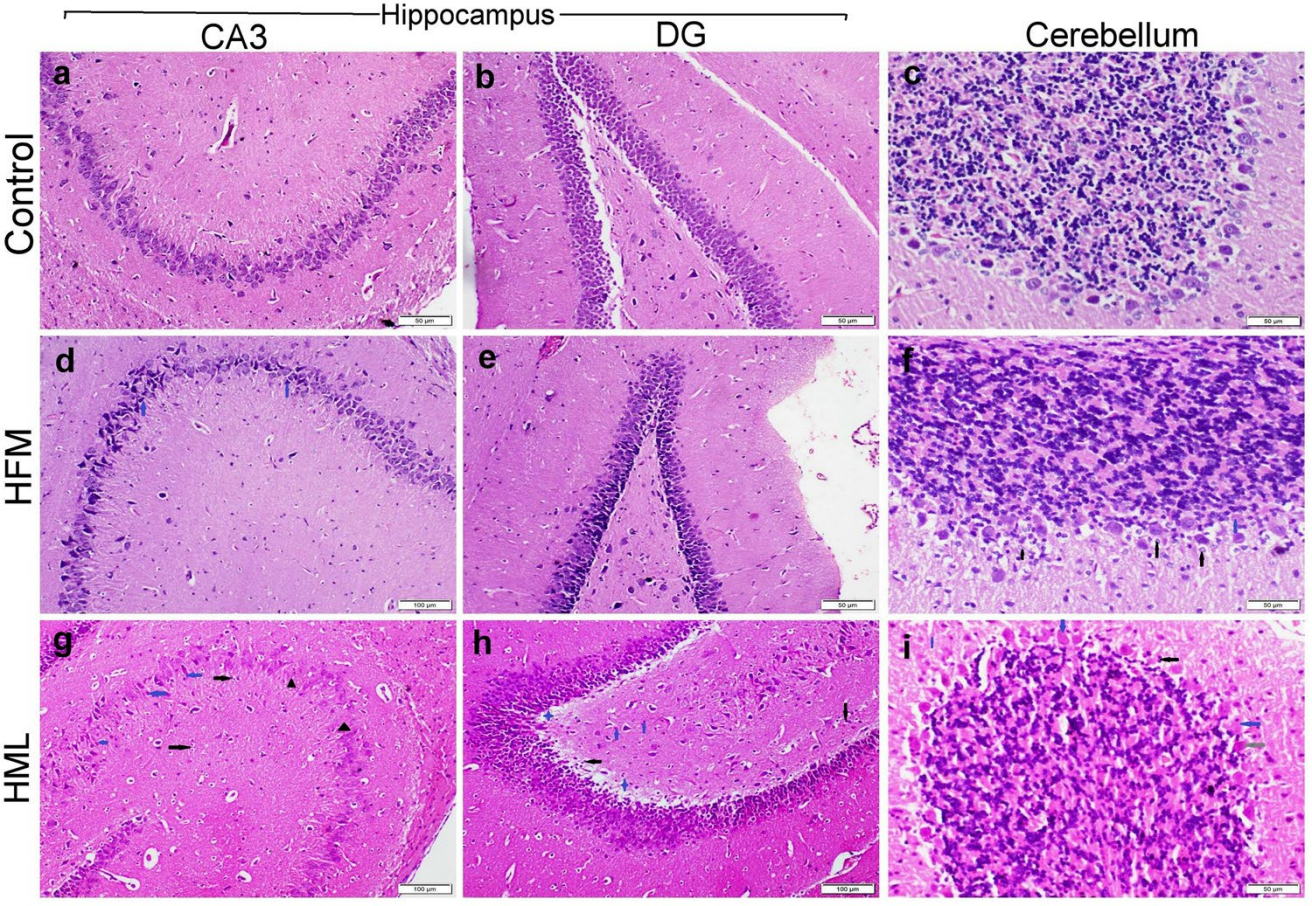
Photomicrograph of H&E staining sections representing cornus ammonis (CA3), dentate gyrus (DG), and cerebellum of different treatment groups. **a**–**c** Control group showed normal histological organization. **d**–**f** HFM receiving group, and **h**–**j** HML receiving group. *Note* Neuronal degeneration (blue arrow), gliosis (black arrow), extravasated RBCs (red arrows), vacuolation (blue star), neuronal loss (black arrowhead)

The hippocampus (Fig. 3a, b), and cerebellum (Fig. 3c) of the control group showed normal histological structures. Likewise, the hippocampus of the HFM group showed the normal histological structure of dentate gyrus (DG) and cornus ammonis layers (CA3) (Fig. 3d, e). Additionally, the cerebellum also showed normal histology of molecular and granular cell layers but with mild vacuolations in the Purkinje layer and sporadic cell necrosis (Fig. 3f). On the other hand, rats exposed to HML showed extensive histopathological alterations in these brain areas. The hippocampus showed vacuolation in the DG (Fig. 3g) and a decrease in the cellular intensity of CA3 layer (Fig. 3h). The cerebellum showed atrophy in the granular layer associated with a decrease in the cellular intensity (Fig. 3i). There was vacuolation in the Purkinje layer with extensive necrosis of Purkinje cells. Cells appeared swollen with eosinophilic cytoplasm and pyknotic or lytic nuclei.

**Fig. 4:**
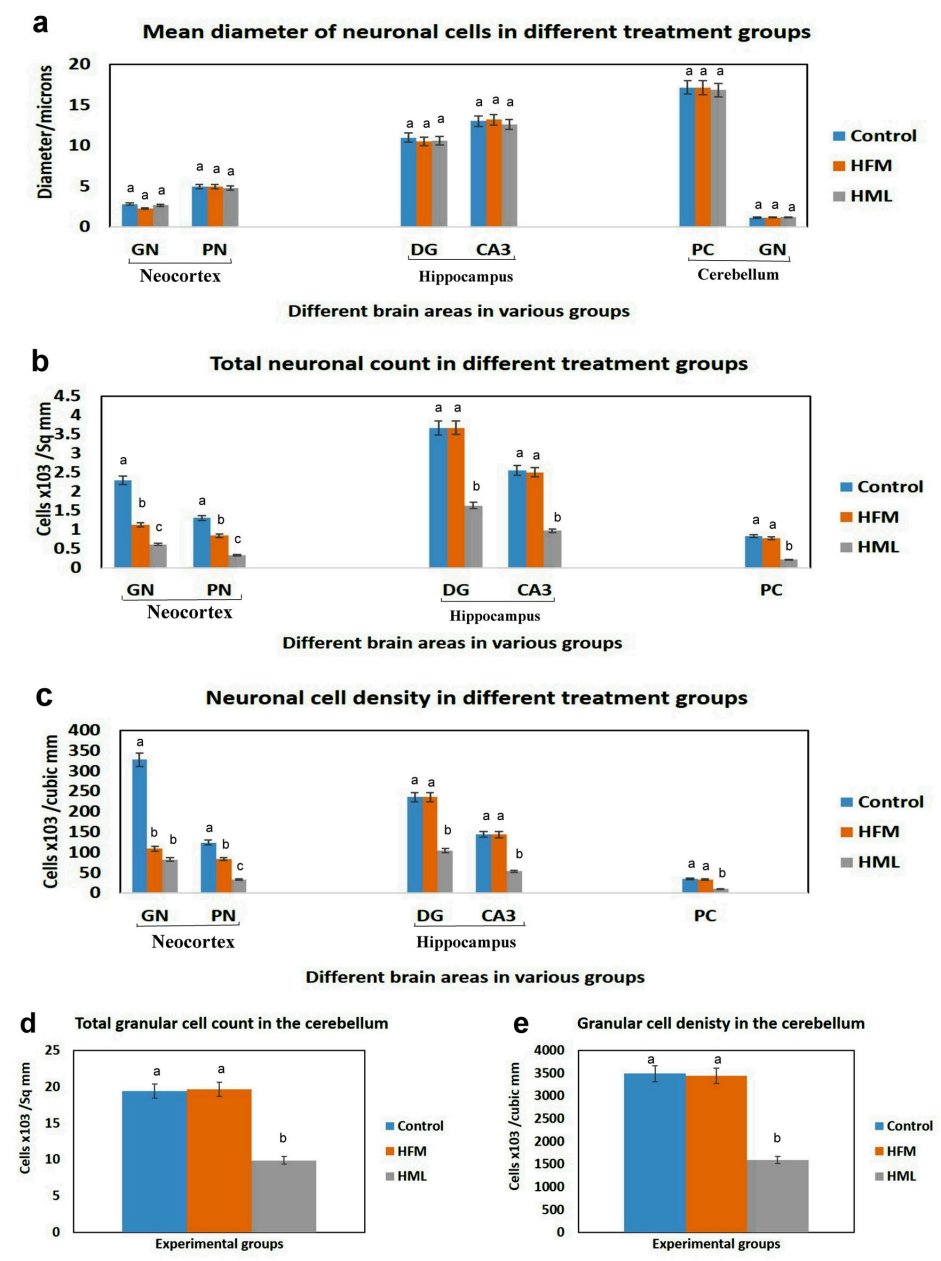
Bar charts representing the histomorphometric analysis of the examined brain areas in various treatment groups. **a** Mean diameter of neuronal cells (microns), **b** total neuronal cell count in mm^2^, **c** absolute neuronal count in mm^3^, **d**, **e** total and absolute neuronal cell count in either mm^2^ or mm^3^ respectively. *Note* Values are presented as mean ± SEM (n = 7 fields/section in total 3 section representing 3 rats/group). Values denoted with different superscript letter (a–c) are statistically significant at P ≤ 0.05. *GN* granular neurons; *PN* pyramidal neurons; *DG* dentate gyrus; *CA3* cornu ammonis; *PC* Purkinje cells

The results of the microscopic lesion scoring were summarized in Table 4. The HML group exhibits a higher score in all parameters in different examined brain areas than the HFM group. Furthermore, the histomorphometric analysis was illustrated in Fig. 4, and noticed that there was no significant difference in neuronal diameter between different treatment groups in the same brain areas, but it differed throughout the various areas. A significant decrease in both neuronal count and density was recorded in the neocortex, DG, CA3, Purkinje cells, and granular cells of the cerebellum obtained from HML receiving. Otherwise, HFM receiving group showed a significant reduction in both neuronal count and density in the neocortex area only compared with the control group but other areas showed normal neuronal count.

**Table 4:** The microscopic lesion scoring in different brain regions

	Control	HFM	HML
Cerebral cortex			
Neuronal degeneration	0^a^	3^b^	4^c^
Gliosis	0^a^	3^b^	4^c^
Hemorrhage	0^a^	2^b^	3^c^
Striatum			
Neuronal loss	0^a^	2^b^	4^c^
Gliosis	0^a^	2^b^	4^c^
Hemorrhage	0^a^	1^b^	3^c^
Hippocampus			
Neuronal degeneration	0^a^	1^b^	4^c^
Gliosis	0^a^	0^a^	2^b^
Hemorrhage	0^a^	0^a^	0^a^
Cerebellum			
Neuronal degeneration	0^a^	1^b^	4^c^
Gliosis	0^a^	0^a^	2^b^
Hemorrhage	0^a^	0^a^	2^b^

Values are presented as median (n = 7 rats/group). Values denoted with different superscript letter (a–c) in the same row are statistically significant at P ≤ 0.05

**Fig. 5:**
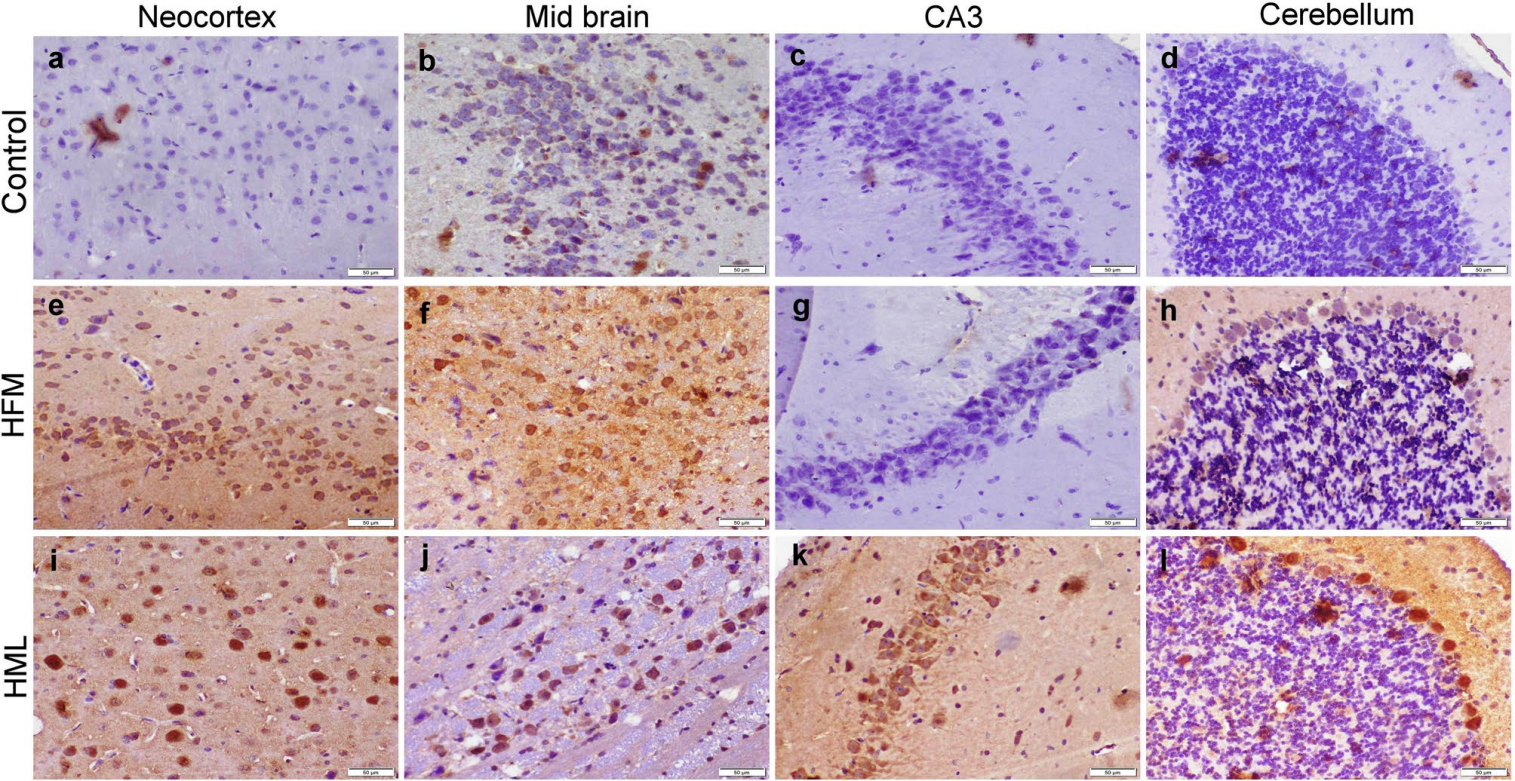
Photomicrograph representing caspase-3 immunostaining in different brain areas of different treatment groups. **a**–**d** The control group showed negative casp-3 expression. **e**–**h** HFM receiving group showed moderate casp-3 expression in the neocortex (**e**) and striatum (**f**) and mild to negative expression in CA3 (**g**), and cerebellum (**h**). **i**–**l** HML receiving group showed strong casp-3 immunopositivity in all the examined areas

### Immunohistochemical Staining

HML exposed group displayed strong positive caspase-3 immunostaining in all the examined brain areas compared with other groups. Meanwhile, HFM receiving group exhibited strong to moderate caspase-3 expression in the neocortex and midbrain compared with the control group, but less than those of the HML group (Fig. 5).

## Discussion

The increasing use of pesticides in agriculture has triggered notable interest in investigating the mechanisms of their neurotoxic impact [20]. Pesticide exposure has been shown to affect a wide range of physiological activities and could be linked to an increased risk of neurological impairment [21–23]. As there is growing evidence that supports the proposed crosstalk between pesticide exposure and neurodegenerative diseases [24, 25]. However, to the best of our knowledge, there is no information available regarding the neurotoxic impact of both HFM and HML pesticides in rats. So, our work is designed to investigate the possible mechanisms participating in both pesticides’ neurotoxicity with comprehensive insights into the role of JNK signaling pathways.

In this study, HML-treated rats showed a decrement in their general activity, as well as, a significant drop in body weight was observed after exposure to HFM and HML regardless of having free access to food, indicating that both pesticides have a toxic effect on animal organs that may hinder the nutrient absorption [26, 27]. Several studies confirmed our findings that insecticides caused weight loss, which could be attributed to the repellant effects of the insecticides causing a reduction in the consumption of food [28, 29]. Behavioral assessment is an essential test for brain functions since changes in the nervous system affect human and animal behavior [30]. In our work, we used the light–dark transition task to evaluate the anxiogenic impacts of the variant agents in rodents [12]. Our findings revealed that HFM and HML induced anxiety-like behavior since rats spent less time in the light compartments whereas, the HML group was more anxious than the HFM group. Furthermore, the rod walking test is another test used for assessing rats' motor activities. As a result, it is an effective practice for determining the degree of locomotor impairment and analyzing the efficacy of elements impacting locomotion and coordination skills including tasks demanding coordinated regulation of motor and reflexive responses, such as how long an animal can traverse/balance on a wooden rod [14]. Based on our findings, exposure of rats to HFM and HML could impair motor coordination and balance but, the marked impairment was recorded in the HML group. The current impairment of the rat’s motor balance could be explained based on HML and HFM-induced histopathological alterations in the substantia nigra and cerebellum since they are responsible for functions including gait, movement, posture, and balance. As a consequence, ataxic movement is one of the most obvious signs of cerebellar dysfunction [31, 32]. A novel object recognition test is a behavioral test that relies on rats’ propensity to explore novel objects. Rats and mice generally prefer to interact with new objects than with familiar objects [33]. Our results revealed a significant increase in the time spent exploring the familiar object in both pesticides treated groups but, the highest time recorded in the HML group. This could be attributed to impairment of visual recognition memory caused by dysfunction of the hippocampus, striatum, and prefrontal cortex in their brain [34–36]. The neurobehavioral results were consistent with the histopathological findings that demonstrate neuronal degeneration and necrosis, gliosis, and intramyelinic edema in various brain areas (cerebrum, striatum, hippocampus, cerebellum) obtained from HFM and HML groups. We believed that these findings attributed to the oxidative stress and mitochondrial dysfunction induced by both pesticides.

Pesticides neurotoxicity can be mediated by oxidative stress through reactive oxygen species (ROS) overgeneration, antioxidant defense depletion, lipid peroxidation, and DNA damage [37]. Based on our findings, both HFM and HML are able to alter the neuronal redox state that is indicated by elevated levels of lipid peroxides (MDA), depletion in GSH content, and TAC in comparison with the control group. Pesticides are proven to induce oxidative stress via several mechanisms including ROS generation, mitochondrial impairments, and their intrinsic redox cycling properties [38]. Moreover, oxidative stress could mediate a variety of pathological conditions including apoptosis, inflammation, and genotoxicity [39, 40].

Apoptosis is a programmed cell death that includes a single or small group of cells [41]. It has occurred via intrinsic or extrinsic pathways. Apoptosis induced by HFM and HMZ has been demonstrated to involve mainly the intrinsic mitochondrial pathway. Both pesticides have a potent pro-oxidant activity that increased the mitochondrial permeability of neurons leading to the release of pro-apoptotic proteins from the mitochondria including cytochrome C which activates caspase cascades ends by activation of caspase-3 that initiate the process of apoptosis [42]. The endoplasmic reticulum (ER) stress is another intrinsic pathway of apoptosis that may or may not depend on the mitochondrial pathway. The ER is very sensitive to stressors that interfere with cellular energy levels, redox states, and Ca^2+^ concentrations leading to the accumulation of toxic unfolded proteins in the cytosol [43, 44]. ER stress leads to activation of c Jun N-terminal kinases (JNKs), which interferes with the anti-apoptotic function of Bcl2, resulting in the activation of pro-apoptotic proteins such as Bax and cell death by caspase activation [45, 46]. Both pesticides can predispose nerve cells to apoptosis by altering the gene signaling pathways that regulate programmed cell death [47, 48]. In order to uncover the underlying mechanism of both HFM and HML neurotoxicity, we selected JNK neuronal signaling pathway. JNKs (c-Jun N-terminal kinases) are a family of protein kinases that play a key role in stress signaling pathways, including gene expression, neural plasticity, regeneration, cell death, and cellular senescence regulation [49]. It has been demonstrated that stressful stimuli, such as cytokines and oxidative stress, activate the JNK pathway which can also phosphorylate and activate apoptosis-related proteins directly such as BIM (homologous to Bax) which led to caspase activation [50, 51]. Also, Bcl-2, are anti-apoptotic proteins that are phosphorylated by JNK [52]. In parallel to our findings, JNK and Bax gene levels were significantly upregulated, and Bcl2 was significantly downregulated in the neural tissue of rats exposed to both HFM and HML. Furthermore, the caspase-3 protein expression was much higher in the examined brain areas of HML group than other groups. Similarly, HFM receiving group displayed strong caspase-3 expression in the neocortex compared with the control group indicating the occurance of apoptosis.

In the current study, we speculated that HML showed more neurotoxic criteria than HFM pesticides in rats. Upon neural redox state evaluation, all oxidative stress parameters (MDA, GSH, and CAT activity) were greatly exacerbated upon exposure to HML than HFM. Consequently, HML showed significant neuronal apoptotic progression (more intensive casp-3 immune reaction) that targets mainly the cerebral cortex, striatum, hippocampus, and cerebellum. when compared to the HFM group that targets the cerebral cortex and striatum only upon histopathological examination. Both pesticides target the JNK signaling pathway that mediates significant neurodegeneration.

## Conclusion

It can be concluded that the exposure to both HFM and HML pesticides led to significant neurobehavioral alterations, neuronal damages, and mitochondrial-mediated apoptotic progression imposed by impaired oxidative status of the brain microenvironment. This altered redox status of the neuronal tissues modulates the JNK signaling pathway and activates the apoptotic pathway. Therefore, the neuropathological mechanism proposed in the current study raises the concern about the strong interplay between exposure to pesticides as mitochondrial neurotoxins and neurodegenerative disorders. However, more strict regulations should be applied to the extensive use of pesticides in the agriculture field for the sake of health and the environment.

## Data Availability

All data are available on request.
